# ESPO/ Academy for Gerontology in Higher Education Section Symposium: Bridging the Gap: Shifting Population Dynamics and Opportunities to Inform and Educate Communities

**DOI:** 10.1093/geroni/igaa057.1743

**Published:** 2020-12-16

**Authors:** Lauren Bouchard, Marilyn Gugliucci

## Abstract

This symposium is intended to highlight population-shifts and their impact on the individual, family, institution, and community levels. Currently, increases in diversity among individuals under age 18 continue to outpace those of individuals age 65-and-older, creating a substantial racial/ethnic diversity gap between generations. As it relates to education-level, individuals age 65-and-older who have earned at least a bachelor’s degree has increased from 5 percent in 1965 to 29 percent in 2018 (Population Reference Bureau, 2020). Population-shifts could be mitigated or better supported through enhancements to gerontology/geriatric education and training. This symposium will highlight the biopsychosocial aspects of these shifts and will link each aspect to the development of gerontology and geriatric curriculum. To start, presenter one will describe projects focused on community-based intergenerational programs to reduce social isolation and loneliness among rural older adults (Jill). Next, presenter two will describe findings from a university-based study, using surveys of aging and ageist attitudes to foster intergenerational connections between undergraduate students and older adult community members (Giselle). Presenter three will discuss a program to support the development of aging ministries to educate and support both caregivers and older adults in community settings (Lauren). Finally, presenter four will discuss findings from one-on-one interviews conducted with Chinese caregivers and Chinese geriatric social workers in efforts to develop and evaluate an end-of-life manual designed for Chinese immigrant caregivers (Mandong). The discussant will link the presenters’ findings to implications for future gerontology and geriatric education and training while identifying key topics to inform and engage communities.

